# Supplementary material for: Structure of substrate-bound SMG1-8-9 kinase complex reveals molecular basis for phosphorylation specificity
Source: eLife. 2020 May 29;9:e57127. doi: 10.7554/eLife.57127 (PMC7334022; doi:10.7554/eLife.57127)
Supplement: Supplementary file 1. [file elife-57127-supp1.docx]

**Supplementary File 1**

| **UPF1_LSQ_-bound SMG1-8-9**  (EMD-11063, PDB ID 6Z3R) | |
| --- | --- |
| **Data collection and processing** | |
| Microscope | FEI Titan Krios GII |
| Voltage (kV) | 300 |
| Camera | Gatan K3 |
| Energy Filter | Gatan Quantum-LS (GIF) |
| Magnification | 81,000x |
| Pixel size (Å/pix) | 1.094 |
| Electron exposure (e^-^/Å^2^) | 68.75 |
| Target defocus range (μm) | 0.5 - 2.5 |
| Number of movies | 6,293 |
| Initially selected particle candidates | 4,368,586 |
| Final number of particles | 481,754 |
| Resolution _FSC independent halfmaps_ (Å) | 2.9 |
| Local resolution range (Å) | 2.8 - 4.5 |
| **Refinement** | |
| Initial model used | 6SYT |
| No. atoms | 36,259 (Hydrogens: 17,260) |
| Residues | 2,636 |
| Ligands |  |
| AMPPNP | 1 |
| ATP | 1 |
| IP6 | 1 |
| Mg | 1 |
| CC_mask,_ CC_box_, CC_peaks_, CC_volume_ | 0.82, 0.78, 0.72, 0.81 |
| Resolution _FSC map vs. model (0/0.143/0.5)_ (Å) | 2.8 / 2.9 / 3.0 |
| *B* factors ( Å^2^) |  |
| Protein | 67.32 |
| Ligand | 55.70 |
| RMS deviations |  |
| Bond lengths (Å) | 0.004 |
| Bond angles (°) | 0.753 |
| **Validation** |  |
| Ramachandran plot |  |
| Favored (%) | 94.15 |
| Allowed (%) | 5.85 |
| Disallowed (%) | 0.00 |
| MolProbity score | 1.42 |
| Clash score | 2.40 |
| Rotamer outliers (%) | 0.00 |
